# A Review of the Relationship Between CTRP Family and Coronary Artery Disease

**DOI:** 10.1007/s11883-020-00840-0

**Published:** 2020-05-28

**Authors:** Yueqiao Si, Wenjun Fan, Lixian Sun

**Affiliations:** Department of Cardiology, The Affiliated Hospital of Chengde Medical University, Chengde, 067000 Hebei China

**Keywords:** Coronary artery disease, Biomarker, Immunology, Inflammation, Metabolism

## Abstract

**Purpose of Review:**

In recent years, a family of adiponectin paralogs designated as C1q/TNF-related protein (CTRP) has attracted increasing attention. They are inflammatory adipocytokines mostly secreted from epicardial adipose tissue, which modulate the development and prognosis of coronary artery disease (CAD). This review summarizes the pathophysiological roles of individual members of the CTRP superfamily in the development of CAD.

**Recent Findings:**

Recent studies have revealed how members of the CTRP family, CTRP1, CTRP3, CTRP5, CTRP9, CTRP12, and CTRP13, can influence both development and progression of CAD by modulating metabolic pathways, influencing immuno-inflammatory response, and regulating cardiovascular functions.

**Summary:**

Research to date has not been sufficient to answer the specific mechanism of the CTRP family in the occurrence and development of CAD. This review explores the evidence of CTRP superfamily regulating different pathophysiology stages of CAD through the immuno-inflammation, glucose and lipid metabolism, and vascular endothelial function.

## Introduction

Endothelial dysfunction, inflammatory response, and metabolic dysregulation are key factors involved in initiation and progression of coronary artery disease (CAD) [1, 2]. Epicardial adipose tissue (EAT) is located inside the pericardial sac, which is adjacent to the epicardium surrounding the heart. EAT is considered a metabolically active organ with endocrine activity. It can secrete a large amount of inflammatory adipocytokines, of which, adiponectin is a well-known cardiovascular protective factor [3, 4]. The C1q complement/tumor necrosis factor (TNF)–associated proteins (CTRPs) superfamily is a paralog of adiponectin, composed of CTRP1-CTRP15, which share a common structural domain with adiponectin [5]. CTRPs mRNA showed highest expression in white adipose tissue around the heart, making it the main secretory organ, though these proteins are also secreted by other viscera, such as the heart and liver [6]. Increasingly, researchers have begun to focus on the pathophysiological role of the CTRP family in cardiovascular diseases. The main members of the CTRP family related to the pathophysiological mechanisms of CAD were found to be CTRP1, CTRP3, CTRP5, CTRP9, CTRP12, and CTRP13. These proteins regulate endothelial function, inflammatory response, and metabolic dysfunction to influence CAD progression.

### Regulation of Immune-Inflammation

CAD is a complex, chronic process that does not simply involve intra-arterial accumulation of cholesterol and calcium. An inflammatory response driven by both pro-inflammatory cells and cytokines also plays an important role in driving development of atherosclerosis and promoting thrombosis, leading to adverse cardiovascular events [7, 8].

CTRP1 regulates low-grade chronic inflammation in coronary atherosclerosis [9]. The inflammatory response and pro-inflammatory cytokines induce increased secretion of CTRP1, which in turn increases expression of adhesion molecules and chemokines such as TNF-α, interleukin (IL)-6, and IL-1b, by activating the p38 MAPK/NF-kB pathway [10–12]. These processes stimulate both in vitro and in vivo adhesion of leukocytes to endothelial cells and further promote formation of macrophages and macrophage-derived foam cells in atherosclerotic plaques, thus exerting a pro-inflammatory, pro-atherogenic effect and accelerating deterioration of CAD [10, 13]. Conversely, CTRP1 can also activate the 1P/cAMP-dependent pathway in cardiomyocytes to reduce apoptosis and inflammatory response, and thus exert a beneficial effect on the pathophysiology of ischemic heart disease [14].

CTRP3 is a potent anti-inflammatory adipokine that inhibits proinflammatory pathways in monocytes and microcells, exerting anti-inflammatory, anti-apoptotic, and cardioprotective effect during development of CAD [15, 16]. CTRP3 affects inhibitory toll-like receptors (TLRs) and nuclear factor kappa B (NF-κB) signaling pathways to reduce secretion of inflammatory adipocytokines, thus attenuating both insulin resistance and obesity-related, chronic, systemic anti-inflammatory responses [17, 18]. Furthermore, CTRP3 promotes activation of the PI3K/Akt/eNOS pathway, which inhibits endothelial inflammation induced by oxidized low-density lipoproteins (ox-LDL) by downregulating expression of CTRP, TNF-α, and IL-6, delaying atherosclerosis progression [19••].

Elevated levels of CTRP5 can promote in-stent restenosis after coronary stent implantation. CTRP5 promotes expression of matrix metalloproteinase-2, cyclin D1, and TNF-α in vascular endothelial cells, by activating Notch1, transforming growth factor (TGF)-β, and hedgehog pathways, thereby promoting the growth, migration, and inflammation of vascular smooth muscle cells (VSMC) [20•].

CTRP9 is the closest paralog of adiponectin, and its anti-inflammatory and anti-atherosclerosis features allow it to play a cardioprotective role in the CAD process [21]. CTRP9 stimulates adenosine monophosphate (AMP)–activated protein kinase pathway to inhibit expression of adhesion molecules such as intercellular adhesion molecule (ICAM)-1 and vascular cell adhesion molecule (VCAM)-1 in endothelial cells, decreases secretion of proinflammatory cytokines such as TNF-α and monocyte chemoattractant protein (MCP)-1 within atherosclerotic plaques, reduces proliferation VSMC, promotes vasodilation (further inhibiting inflammatory responses in macrophages), and thus increases stability of atherosclerotic plaques [22, 23•, 24].

CTRP12 is associated with inflammatory cytokines and plays a potential role in atherosclerosis. CTRP12 is known to reduce the expression of pro-inflammatory cytokines and decrease macrophage accumulation within adipose tissue in obese mice, and also was found to inhibit the secretion of inflammatory cytokines IL-6 and TNF-α in CAD patients [25, 26]. Therefore, overexpression of CTRP12 exerts an anti-inflammatory effect during both development and deterioration of CAD.

CTRP13 inhibits macrophage activation and infiltration of vessel walls, reduces plaque formation, and thereby inhibits development of atherosclerosis. Furthermore, it prevents proliferation and migration of macrophages by downregulating lipid uptake, delays local and systemic inflammatory responses during atherosclerosis by promoting autophagy (in macrophages), and accelerates CD36-dependent degradation of autophagolysosomal pathways, thus reducing number of macrophages in lesions [27, 28]. A case-control study found that CTRP13 led to decreases in obesity and inflammation and that it showed negative correlation with proinflammatory cytokines such as TNF-α and IL-6, while TNF-α and body mass index (BMI) were its independent negative predictors [16].

### Effects on Glucose and Lipid Metabolism

Glucose and lipid metabolism are the two major processes involved in increasing the risk and severity of CAD. Abnormal metabolism affects activity of regulatory pathways, composition of the final product, degree of inflammation, and coronary-plaque formation, thus contributing to the development of CAD and accelerating occurrence of adverse cardiovascular events [29, 30].

CTRP1 is involved in regulation of obesity-related, metabolic, and cardiovascular diseases, and affects cardiac metabolism by primarily regulating blood glucose and lipid metabolism [31]. Increased expression of CTRP1 could improve insulin sensitivity and glucose tolerance, which in turn may increase glucose metabolism and reduce adiposity in an overnutritional state [32]. CTRP1 increases fatty acid oxidation and energy expenditure. It inhibits acetyl-CoA carboxylase (ACC) via the AMP protein kinase (AMPK) pathway to attenuate obesity [33].

CTRP3 is a cardioprotective, anti-inflammatory cytokine. It improves insulin sensitivity, enhances insulin-mediated glucose uptake, and reduces hepatocyte gluconeogenesis (and subsequent glucose output), thereby slowing down development of CAD [34, 35]. A cross-sectional study found low levels of CTRP3 in association with CAD, though its levels in correlation to obesity and diabetes showed sex-specific differences [36].

CTRP5 upregulates 12/-15-lipoxygenase (LOX) expression via the signal transducer and activator of transcription (STAT)-6 signaling pathway. Inhibition of the STAT6-12/15-lipoxygenase-dependent pathway attenuates CTRP5-induced transcytosis and oxidative modification of the LDL transendothelial monolayers, thereby retarding development and progression of early-stage atherosclerosis [37].

CTRP9 regulates lipid metabolism and enhances the AMPK/mTOR autophagy signaling pathway to enhance acid-lipid-mediated cholesterol efflux, increases the level of expression of the cholesterol-transporting receptors like ATP-binding membrane cassette transporter (ABC) A1, and G1 (ABCG1), which accelerates cholesterol efflux from foam cells, thereby preventing THP-1 macrophages from forming foam cells and slowing progression of early atherosclerosis [24, 38]. An in vivo study of mice showed that overexpression of CTRP9 reduced fasting insulin levels and fasting blood glucose, increasing insulin sensitivity [39, 40]. CTRP9 correlated positively with parameters of glucose metabolism by activating Akt, AMPK, and p42/44 MAPK pathways, and further increasing glucose uptake [41].

CTRP12 inhibits gluconeogenesis and increases glucose uptake in hepatocytes and adipocytes by activating the PI3K-Akt signaling pathway and improving insulin sensitivity [42]. CTRP12 exerts a beneficial effect on glucose and insulin metabolism and plays a potential detrimental role in atherosclerosis via its association with insulin resistance, decreased high-density lipoprotein cholesterol, and increased BMI [43, 44].

CTRP13 exerts a beneficial effect during insulin-mediated glucose uptake, which can reduce glucose output in hepatocytes by inhibiting the expression of gluconeogenic enzymes. CTRP13 reduces phosphorylation of AMPK in adipocytes, muscle cells, and hepatocytes; promotes AMPK signaling pathway activation to increase glucose uptake in adipocytes; and inhibits stress-activated protein kinase/JNK stress signaling, to decrease lipid-induced, insulin resistance in hepatocytes, thereby reducing hepatocytic gluconeogenesis and decreasing insulin resistance [34]. In addition, CTRP13 hydrolyzes cholesterol droplets stored in macrophages, inhibits intracellular influx of cholesterol, and promotes cholesterol efflux, thus inhibiting the formation of foam cells and decelerating progression of atherosclerosis [45, 46].

### Mechanisms of Vascular and Myocardial Injury

Coronary artery endothelial injury is an early event in the pathological process of atherosclerosis, mediated via immune-inflammation, oxidative stress, and biochemicals [47, 48]. Endothelial cells are constantly exposed to these stimulating factors and strive to maintain antithrombotic, anti-inflammatory, and anti-proliferative homeostasis through compensatory responses [49]. This normal homeostasis, when impaired, can aggravate subsequently the inflammatory response, leading to vasomotor dysfunction and ventricular remodeling, as seen after myocardial infarction [50, 51].

CTRP1 may serve as a vasculoprotective adipokine, with similar effects on vascular injury as seen with adiponectin. Increased expression of CTRP1 reduces neointimal hyperplasia and cell proliferation in damaged arteries after vascular injury, through inhibition of VSMC growth via cAMP-dependent pathways. In addition, it attenuates accumulation of macrophages in damaged blood vessels, while leaving the degree of both apoptosis and reendothelialization unaffected [52, 53]. CTRP1 prevents pathological vascular remodeling, inhibits formation of collateral circulation by inhibiting in vitro angiogenesis of endothelial progenitor cells, and prevents vascular stenosis after injury [54]. CTRP1 is an acute phase reactant of inflammation and is positively associated with fibrinogen, which can cause cross-linking and aggregation of platelets, leading to thrombosis, thereby indicating an association with adverse cardiovascular events [55].

CTRP3 reduces myocardial damage after ischemia and plays a cardioprotective role. CTRP3 attenuates pathological myocardial remodeling after an acute infarction through inhibition of myocardial fibrosis and enhances the survival and regeneration of ischemic cardiomyocytes [56, 57]. In addition, CTRP3 can possibly increase Akt phosphorylation and induce expression of hypoxia-inducible factor 1-α, vascular endothelial growth factor, and promote secretion of angiogenic factors from endothelial cells, which can contribute to angiogenesis [56].

CTRP9 has a higher vasoactive potency than adiponectin and plays an important role in the regulation of vascular stiffness [58]. It can promote vasodilation, inhibit both neointimal hyperplasia and endothelium-dependent VSMC proliferation, attenuate atherosclerosis, and exert a protective effect on cardiac remodeling after acute myocardial infarction [59–61]. Overexpression of CTRP9 in circulation and in EAT was found to significantly attenuate VSMC proliferation and neointimal formation [62].

## Conclusion

**Fig. 1:**
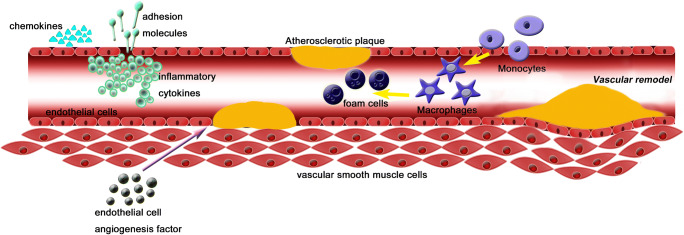
Macrophages derive from monocytes, which form foam cells following phagocytosis of lipids. The accumulation of macrophages and foam cells promotes the formation of atherosclerotic plaques. Vascular injury promotes secretion of inflammatory cytokines, adhesion molecules, and chemokines, which aggravates the inflammatory response of the vascular endothelium and promotes plaque formation. Endothelial cell angiogenic factors promote endothelial cell proliferation and enhance the survival and regeneration of ischemic cardiomyocytes. Vascular remodeling due to long-term chronic inflammation stimulation manifests as the thickened blood vessel wall and the narrowed lumen

The CTRP family plays an important role in all stages of CAD by regulating immuno-inflammation, glucose and lipid metabolism, and vascular endothelial function. (Table 1) CTRP1 represents as pro-inflammatory and pro-atherosclerotic markers by contributing toward the secretion of inflammatory cytokines and adhesion molecules and promoting the formation of foam cells from macrophages. CTRP5 promotes VSMC growth, migration, and inflammation. In contrast, CTRP3, CTRP9, CTRP12, and CTRP13 activate anti-inflammatory and anti-atherosclerotic mechanisms of CAD, by inhibiting endothelial inflammation and reducing plaque formation (mediated via inhibition of both inflammatory cytokine secretion and expression of adhesion molecules). Also, these four family members reduce macrophage accumulation and foam-cell formation. CTRP family members regulate vascular endothelial inflammation and plaque formation by regulating glucose and lipid metabolism. This protein superfamily could improve insulin sensitivity, decrease insulin resistance, increase glucose tolerance, enhance glucose uptake, and reduce gluconeogenesis. Furthermore, they also enhance expression of cholesterol transport receptors, promote cholesterol efflux, and increase fatty acid oxidation. CTRP1, CTRP3, and CTRP9 increase expression of HIF1α and vascular endothelial growth factor, promote secretion of endothelial cell angiogenic factors, inhibit neointimal hyperplasia and VSMC proliferation, and inhibit myocardial fibrosis, thus supporting the survival and regeneration of ischemic cardiomyocytes (Fig. 1).

**Table 1:** Summary of CTRP and their potential mechanism on CAD

	Search phrase and numbers	Distribution	Signaling pathway	Inflammatory function	Metabolic function	Endothelial injury function		Relationship between CTRP and CAD	Reference
CTRP1	CTRP1 AND CAD (6)	CTRP1 AND coronary (18)	Adipose tissue, heart, placenta, liver, kidney, muscle, prostate, ovary, etc.	p38 MAPK/NF-kB pathway; AMPK pathway; cAMP-dependent pathway	Promote the expression of adhesion molecules and chemokines; promote the formation of macrophage foam cells	Improve insulin sensitivity and glucose tolerance; enhance fatty acid oxidation and energy expenditure	Inhibit VSMC growth and angiogenesis in vitro	CTRP1 levels were increased in CAD patients and increased with increase in severity of CAD.	[10–12, 14, 33, 56, 58]
CTRP3	CTRP3 AND CAD (3)	CTRP3 AND coronary (11)	Adipose tissue, kidney, ovary, brain, monocytes, colon, fibroblasts, placenta, pancreas, etc.	PI3K/Akt/eNOS pathway; NF-κB pathways	Inhibit the secretion of inflammatory adipocytokines; inhibit endothelial inflammatory responses	Improve insulin sensitivity and glucose uptake; reduce hepatocyte gluconeogenesis	Promote the secretion of endothelial cell angiogenesis factors and angiogenesis; inhibit myocardial fibrosis; enhance the survival/ regeneration of ischemic cardiomyocytes	CTRP3 levels were decreased in CAD patients and negatively correlated with an increased risk of CAD.	[16, 19••, 36, 62]
CTRP5	CTRP5 AND CAD (1)	CTRP5 AND coronary (7)	Adipose tissue, brain, myocytes, basement membrane, etc.	Notch1,TGF-β and hedgehog pathways; STAT6 signaling pathway; STAT6-12/15-lipoxygenase-dependent pathway	Promote the inflammation of vascular smooth muscle cells	Induced transcytosis and oxidative modification of low-density lipoprotein transendothelial and promote early-stage atherosclerosis	Promote the growth and migration of vascular smooth muscle cells	CTRP5 levels were increased in CAD patients and positively correlated with the number of diseased vessels.	[20•, 37]
CTRP9	CTRP9 AND CAD (3)	CTRP9 AND coronary (21)	Adipose tissue; stromal vascular cells	AMP-activated protein kinase pathway; Akt, AMPK and p42/44 MAPK pathway; AMPK/mTOR autophagy pathway	Inhibit the expression of adhesion molecules and the secretion of pro-inflammatory cytokines; reduce the formation of macrophage foam cells	Improve insulin sensitivity; enhance the expression of the cholesterol transport receptors and cholesterol efflux	Inhibit vascular smooth muscle cell proliferation and neointimal hyperplasia	CTRP9 levels and mRNA expression were decreased in CAD patients and is an independent protective factor of CAD.	[21–24, 41]
CTRP12	CTRP12 AND CAD (1)	CTRP12 AND coronary (4)	Adipose tissue, kidney, spleen, uterus	PI3K-Akt pathway	Inhibit the secretion of inflammatory cytokines; reduce macrophage accumulation and plaque formation	Improve insulin sensitivity; inhibit gluconeogenesis	_	CTRP12 levels were decreased in CAD patients is independent associated with CAD and several CAD risk factors.	[25]
CTRP13	CTRP13 AND CAD (1)	CTRP13 AND coronary (3)	Adipose tissue, brain, kidney	Autophago lysosomal-dependent pathways; AMPK pathway; protein kinase/JNK stress pathway	Inhibit inflammatory cytokines; inhibit macrophage infiltration and activation; inhibit the formation of foam cells and plaque formation	Improve insulin resistance; reduce hepatocyte gluconeogenesis; inhibit the influx of cholesterol and promotes cholesterol efflux	_	CTRP13 levels were decreased in CAD patients and are negatively associated with an increased risk of CAD.	[27]

CTRP1 and CTRP5, as possible risk factors for CAD, elevate in patients with CAD and associate with the severity of coronary stenosis. On the contrary, CTRP3, CTRP9, CTRP12, and CTRP13, as protective factors for CAD, decrease in patients with CAD. Thereby, this review on CTRP superfamily may take unique insight into the development and progression of CAD. Positive results from such research and further understanding of their molecular mechanisms will promote adding these biomarkers to CAD diagnostic guidelines.

## Abbreviations

CAD: Coronary artery disease
EAT: Epicardial adipose tissue
TNF: Tumor necrosis factor
CTRPs: C1q complement/tumor necrosis factor (TNF)–associated proteins
TLR: Toll-like receptor
NF-κB: Nuclear factor kappa B
Ox-LDL: Oxidized low-density lipoproteins
IL: Interleukin
MMP: Matrix metalloproteinase
TGF: Transforming growth factor
AMP: Adenosine monophosphate
ICAM: Intercellular adhesion molecule
VCAM: Vascular cell adhesion molecule
MCP: Monocyte chemoattractant protein
VSMC: Vascular smooth muscle cells
BMI: Body mass index
AMPK: AMP protein kinase
ACC: Acetyl-CoA carboxylase
LOX: Lipoxygenase
STAT: Signal transducer and activator of transcription
ABC: ATP-binding membrane cassette transporter
